# Mitogenomic Evidence for the Phylogenetic Placement of *Chimarrichthys kishinouyei* Within Sisoridae

**DOI:** 10.3390/genes17070749

**Published:** 2026-06-29

**Authors:** Ping Ying, Ting Yang, Zhihua Lin, Jie Chen

**Affiliations:** 1 Industrial College of Traditional Chinese Medicine and Health, Lishui University, Lishui 323000, China; ping@lsu.edu.cn (P.Y.); linzhihua1015@126.com (Z.L.); 2 College of Agriculture and Biotechnology, Lishui University, Lishui 323000, China; yangting001210@163.com

**Keywords:** *Chimarrichthys kishinouyei*, mitochondrial genome, Sisoridae, codon usage, phylogenetic analysis

## Abstract

**Background**: The phylogenetic placement of the rheophilic glyptosternoid catfish *Chimarrichthys kishinouyei* within Sisoridae remains insufficiently resolved because mitogenome-derived phylogenetic evidence has been unavailable. **Methods**: We sequenced, assembled, and annotated the complete mitogenome of *C. kishinouyei* and reconstructed its mitochondrial phylogenetic relationships using 13 protein-coding genes and two rRNA genes. **Results**: The mitogenome was a circular molecule of 16,718 bp and contained 37 typical mitochondrial genes, including 13 protein-coding genes, 22 tRNA genes, two rRNA genes, and a control region. The genome showed an A + T bias, with an A + T content of 57.27%. Most tRNAs formed typical cloverleaf structures, whereas tRNA-Ser(GCU) lacked a typical DHU arm. Codon usage was biased, and all 13 protein-coding genes had Ka/Ks ratios below 1, consistent with predominant purifying selection. Phylogenetic analyses placed *C. kishinouyei* within the glyptosternoid lineage and recovered a strongly supported sister relationship with *Pareuchiloglanis sichuanensis* rather than direct clustering with *Euchiloglanis davidi*. **Conclusions**: Phylogenetic analyses based on 13 protein-coding genes and two rRNA genes support the placement of *C. kishinouyei* within glyptosternoid Sisoridae and indicate that relationships among *Chimarrichthys*, *Pareuchiloglanis*, and *Euchiloglanis* require further testing with broader integrative evidence.

## 1. Introduction

Animal mitochondrial genomes are compact, usually maternally inherited, and generally conserved in gene content and organization. In most animals, the mitochondrial genome is a circular molecule of approximately 16 kb and typically contains 37 genes, including 13 protein-coding genes (PCGs), 22 transfer RNA genes, and two ribosomal RNA genes [1]. Because of their small size, conserved organization, and relatively rapid evolutionary rate, mitochondrial genomes have been widely used in species identification, comparative genomics, population genetic studies, and phylogenetic reconstruction. Compared with single mitochondrial markers, mitogenome-scale datasets provide a larger number of informative characteristics and are therefore valuable for resolving relationships among closely related fish taxa [1,2].

Recent comparative fish mitogenome studies have shifted complete mitochondrial data from genome reporting toward lineage-level phylogenetic interpretation. By comparing genome size, gene order, strand-specific nucleotide composition, codon usage, tRNA secondary structures, control-region variation, and mitochondrial tree topology, these studies have shown that conserved mitochondrial organization can provide a stable framework for assessing lineage-specific sequence variation and matrilineal signals [3,4,5,6]. This comparative perspective is especially relevant for rheophilic and taxonomically complex freshwater fishes, in which morphological convergence and incomplete taxon sampling can obscure relationships inferred from phenotype or short mitochondrial markers.

Sisoridae is a diverse family of Asian catfishes, many members of which inhabit torrential streams and mountain rivers. These fish often possess specialized adaptations to fast-flowing environments, including depressed bodies, expanded paired fins, and adhesive structures that facilitate attachment to substrates in strong currents [7,8]. Despite their ecological and morphological diversity, phylogenetic relationships within Sisoridae remain difficult to resolve, especially among glyptosternoid genera adapted to mountain streams. Previous studies based on morphology or limited mitochondrial markers, such as *cytochrome b* and *16S rRNA*, have clarified several major relationships but have left some intrafamilial and intergeneric affinities uncertain [8,9,10]. Thus, additional mitogenome assemblies and homologous mitochondrial gene datasets are needed for testing taxonomic hypotheses and evaluating evolutionary relationships in this group.

*Chimarrichthys kishinouyei* (Kimura, 1934) is a rheophilic glyptosternoid sisorid catfish distributed in the upper Yangtze River basin of China. This species was historically treated as *Euchiloglanis kishinouyei* and is currently recognized within *Chimarrichthys*, reflecting the complex taxonomic history of glyptosternoid catfishes [11,12]. Members of this lineage are closely associated with torrential freshwater habitats, and previous morphometric and population genomic analyses of the *Chimarrichthys* fish complex revealed substantial phenotypic and genomic divergence on the Tibetan Plateau [13]. This taxonomic background makes *C. kishinouyei* a useful case for testing whether mitochondrial genomic evidence supports its current placement and for assessing its affinity to other glyptosternoid genera. However, the mitochondrial genome of *C. kishinouyei* has not been formally described. This gap limits phylogenetic assessment of its systematic position within Sisoridae.

In light of the recent phylogenetic and classification framework for Sisoridae [14], we used a concatenated mitochondrial dataset of 13 PCGs and two rRNA genes to test the phylogenetic placement of *C. kishinouyei* within Sisoridae. Specifically, we sequenced, assembled, and annotated the complete mitochondrial genome of a newly sampled individual, characterized genomic features relevant to mitochondrial sequence evolution, and reconstructed relationships using a concatenated dataset of 13 PCGs and two rRNA genes under maximum likelihood and Bayesian inference frameworks. This approach places mitogenome characterization within a phylogenetic framework and provides new mitochondrial evidence for evaluating the placement of *C. kishinouyei* within the glyptosternoid lineage.

## 2. Materials and Methods

### 2.1. Sample Collection and Species Identification

**Figure 1:**
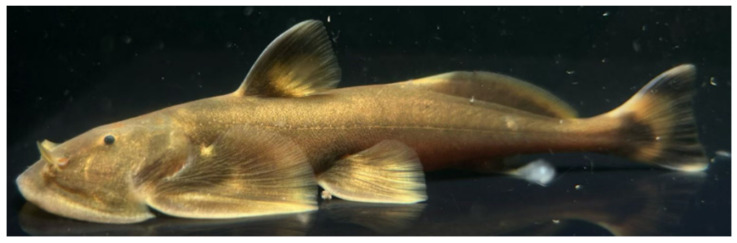
Specimen of the *Chimarrichthys kishinouyei* used in this study.

An adult specimen of *C. kishinouyei* was collected from the middle-upper reaches of the Mengxi River, a tributary of the Tuojiang River, near Leyangqiao Village, Lezhi County, Ziyang City, Sichuan Province, China (30.0465° N, 105.0389° E). After sampling, the specimen was rinsed with clean water to remove surface contaminants. Muscle tissue was dissected from the specimen, immediately preserved in absolute ethanol, and subsequently stored at −80 °C until DNA extraction. Species identification was performed using external morphological characteristics described in previous taxonomic treatments of glyptosternoid sisorid catfishes [11,12]. The specimen was photographed before tissue sampling (Figure 1).

### 2.2. DNA Extraction and Sequencing

Total genomic DNA was isolated from the preserved muscle tissue with the Rapid Animal Genomic DNA Isolation Kit (Sangon Biotech, Shanghai, China), following the manufacturer’s instructions. DNA integrity was evaluated by 1% agarose gel electrophoresis, and DNA concentration was quantified using a Qubit fluorometer (Thermo Fisher Scientific, Waltham, MA, USA). A genomic DNA library with an insert size of 350 bp was prepared using the TruSeq Nano DNA Library Prep Kit (Illumina, San Diego, CA, USA). The library was then sequenced on the Illumina NovaSeq X Plus platform (Illumina, San Diego, CA, USA) to generate 150 bp paired-end reads.

### 2.3. Mitogenome Assembly and Annotation

Raw reads were processed with fastp v0.20.0 [15] to remove adapters and low-quality sequences, producing high-quality clean reads. Because sequencing was performed from total genomic DNA, mitochondrial reads were not separated experimentally before sequencing. Instead, the clean-read dataset was assembled de novo using SPAdes v4.2.0 [16] in paired-end mode with default parameters, and k-mer sizes were automatically selected by SPAdes. Candidate mitochondrial contigs were screened from the assembly by comparison with closely related sisorid mitogenomes and by the presence of the typical vertebrate mitochondrial gene set. Clean reads were then mapped back to the candidate mitochondrial contig in Geneious Prime 2025.0.2 to verify read support, circularity, and coverage continuity. To reduce the risk of contamination and nuclear mitochondrial DNA segments (NUMTs), the final mitogenome was retained only after confirming that it represented a single circular molecule, contained the expected 37 mitochondrial genes, showed no obvious assembly breaks or conflicting read support, and had intact protein-coding genes without unexpected internal stop codons or frameshifts. The assembled mitochondrial genome was annotated with MITOS2 [17] under the vertebrate mitochondrial genetic code. Gene boundaries were manually checked and adjusted in Geneious Prime 2025.0.2 by comparison with homologous genes from closely related sisorid mitogenomes. tRNA genes and their putative secondary structures were further examined using the tRNAscan-SE web server [18].

### 2.4. Sequence Analyses

Nucleotide composition was calculated for the whole mitogenome and for major genomic regions using Geneious Prime 2025.0.2. Strand asymmetry was estimated using the formulas AT-skew = [A − T]/[A + T] and GC-skew = [G − C]/[G + C] [19]. Amino acid composition and relative synonymous codon usage (RSCU) values of the PCGs were calculated using PhyloSuite v2 [20] and visualized with an R script. For PCG-level nucleotide diversity and Ka/Ks analyses, we used the Sisoridae ingroup dataset listed in Table S1, comprising the newly assembled mitogenome of *C. kishinouyei* and 47 published Sisoridae mitogenomes. The 13 orthologous PCGs were extracted from each mitogenome using PhyloSuite v2 [20], aligned separately with MAFFT v7.313 [21], refined with MACSE v2.03 [22] to maintain codon-aware alignment and reading-frame consistency, and trimmed with Gblocks v0.91b [23] to remove poorly aligned regions. For nucleotide diversity analysis, the aligned and trimmed PCGs were concatenated and imported into DnaSP v6.12.03 [24]. Nucleotide diversity (π) was calculated across this concatenated PCG alignment and plotted by gene region to compare PCG-level sequence variation among Sisoridae. For Ka/Ks analysis, each aligned and trimmed PCG dataset was analyzed separately in DnaSP v6.12.03 [24], and Ka, Ks, and Ka/Ks values were summarized by gene. Alignment matrix statistics for the PCG datasets are provided in Table S2.

### 2.5. Phylogenetic Analyses

The newly assembled mitogenome of *C. kishinouyei* and 47 additional mitochondrial genomes of Sisoridae retrieved from NCBI were used as ingroup taxa, and three mitochondrial genomes of Bagridae were selected as outgroups (Table S1). All phylogenetic analyses were conducted in PhyloSuite v2 [20]. The 13 PCGs and two rRNA genes were selected because they are homologous mitochondrial regions shared by all sampled taxa and can be aligned more reliably than whole mitochondrial genome alignments, which include highly variable non-coding regions such as the control region. Separating PCGs and rRNAs also allowed codon-aware alignment of PCGs and independent alignment and trimming of rRNA genes. The 13 PCGs were aligned using MAFFT v7.313 [21] and further refined with MACSE v2.03 [22] to maintain codon-aware alignments. Poorly aligned positions and divergent regions were removed with Gblocks v0.91b [23]. The two rRNA genes were aligned using MUSCLE v5.3 [25], and ambiguously aligned regions were filtered with trimAl v2 [26]. The aligned 13 PCGs and two rRNA genes were concatenated into a single supermatrix using PhyloSuite v2.

ModelFinder [27] was used to determine the best-fit partition schemes and substitution models for maximum likelihood (ML) and Bayesian inference (BI) analyses based on the corrected Akaike information criterion (AICc). Although the PCGs and rRNA genes provide biologically meaningful initial partitions, the best-fit partition scheme was used to allow ModelFinder to merge partitions with similar evolutionary patterns and to reduce unnecessary model complexity. The selected partition schemes and substitution models for ML and BI analyses are provided in Table S3. The ML tree was reconstructed using IQ-TREE v3.1.1 [28], with nodal support evaluated by 10,000 ultrafast bootstrap replicates. BI analysis was performed in MrBayes v3.2.7a [29] with two independent runs, each consisting of four Markov chains for five million generations. Trees were sampled every 1000 generations, and the first 25% of samples were discarded as burn-in. Convergence was considered adequate when the average standard deviation of split frequencies fell below 0.01. Phylogenetic trees were visualized using the iTOL online platform [30].

## 3. Results

### 3.1. Mitogenome Organization and Nucleotide Composition

**Figure 2:**
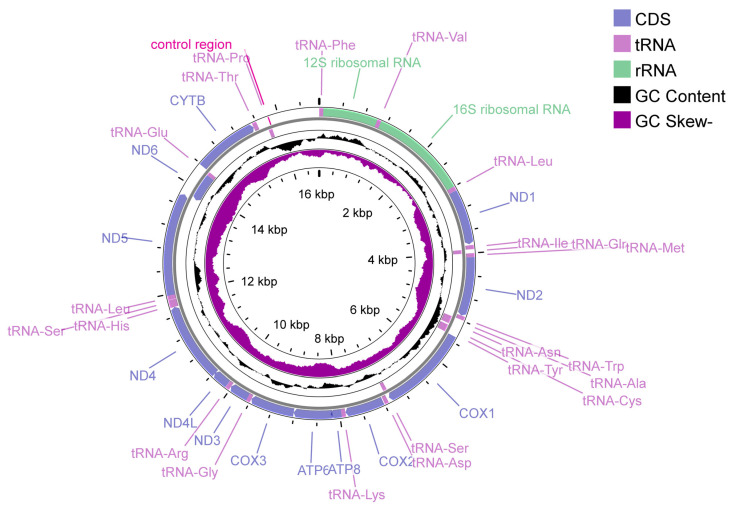
Gene map of the complete mitochondrial genome of *C. kishinouyei*.

The complete mitochondrial genome of *C. kishinouyei* was a circular molecule of 16,718 bp (Figure 2). It contained 37 typical mitochondrial genes, including 13 PCGs, 22 tRNA genes, two rRNA genes, and a putative control region (D-loop) (Table S4). The gene arrangement was consistent with the common teleost mitochondrial gene order. Most genes were encoded on the heavy strand, whereas *ND6* and eight tRNA genes were located on the light strand. Among the 13 PCGs, *ATP8* was the shortest gene (189 bp), while *ND5* was the longest (1827 bp). The *12S rRNA* and *16S rRNA* genes were 952 bp and 1703 bp in length, respectively, and were positioned between *tRNA-Phe* and *tRNA-Leu*, separated by *tRNA-Val*. The 22 tRNA genes ranged from 67 bp to 77 bp. The control region was located between *tRNA-Pro* and *tRNA-Phe* and was 893 bp long.

The nucleotide composition of the whole mitogenome was A = 31.79%, T = 25.48%, G = 15.26%, and C = 27.47%, resulting in an overall A + T content of 57.27% (Table 1). The mitogenome had a positive AT-skew value of 0.11 and a negative GC-skew value of −0.29, indicating a higher proportion of A than T and a higher proportion of C than G. Among the analyzed genomic regions, the D-loop showed the highest A + T content (62.26%).

### 3.2. Protein-Coding Genes and Codon Usage

**Figure 3:**
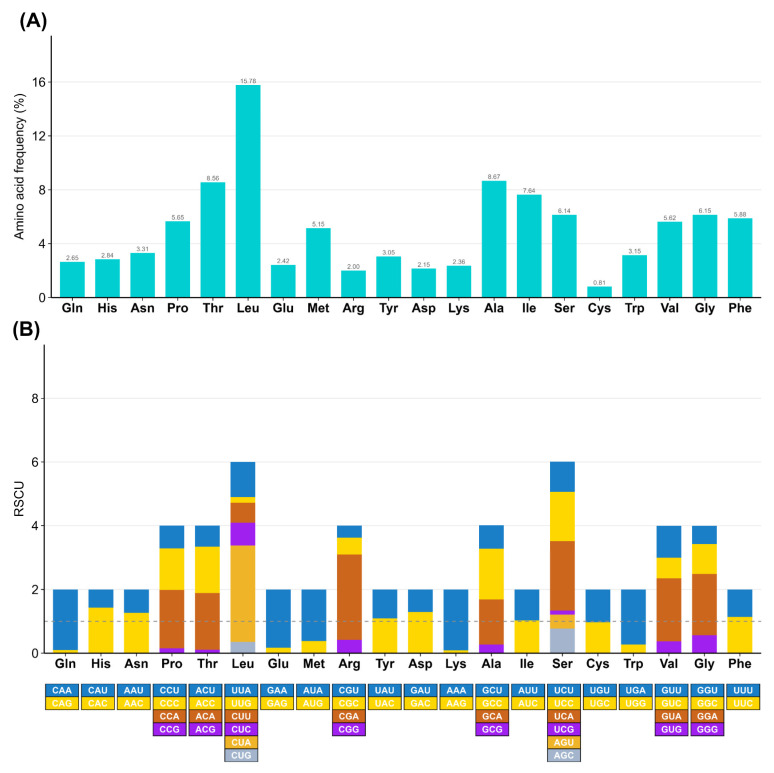
Amino acid composition and relative synonymous codon usage in the 13 mitochondrial PCGs of *C. kishinouyei*. (**A**) Amino acid usage; (**B**) RSCU values. The dashed line indicates RSCU = 1.

Amino acid usage in the 13 mitochondrial PCGs of *C. kishinouyei* is summarized in Figure 3A. Leu was the most abundant amino acid, accounting for 15.78% of the total amino acid composition, followed by Ala (8.67%), Thr (8.56%), Ile (7.64%), Gly (6.15%), Ser (6.14%), Phe (5.88%), Pro (5.65%), Val (5.62%), and Met (5.15%). By contrast, Cys was the least frequent amino acid, accounting for only 0.81%.

RSCU analysis revealed uneven use of synonymous codons among the 13 PCGs (Figure 3B). Several codons showed RSCU values greater than 1, indicating a clear codon usage bias. The preferentially used codons included CUA for Leu, GCC for Ala, ACA for Thr, CCA for Pro, AUC for Ile, GGA for Gly, and UUC for Phe. Overall, the codon usage profile was consistent with the nucleotide composition bias of the mitogenome.

### 3.3. Transfer and Ribosomal RNA Genes

Twenty-two tRNA genes were detected in the mitogenome of *C. kishinouyei*, with lengths ranging from 67 bp to 77 bp (Table S4). As shown in Figure S1, most tRNAs were predicted to fold into typical cloverleaf structures composed of the acceptor stem, TΨC arm, anticodon arm, variable loop, and DHU arm. In contrast, tRNA-Ser(GCU) lacked a typical DHU arm and formed an incomplete cloverleaf structure. Two tRNA-Leu genes, tRNA-Leu(UAA) and tRNA-Leu(UAG), and two tRNA-Ser genes, tRNA-Ser(GCU) and tRNA-Ser(UGA), were identified. The *12S rRNA* and *16S rRNA* genes were 952 bp and 1703 bp in length, respectively, and were located between tRNA-Phe and tRNA-Leu(UAA), separated by tRNA-Val.

### 3.4. Selective Pressure Analysis

**Figure 4:**
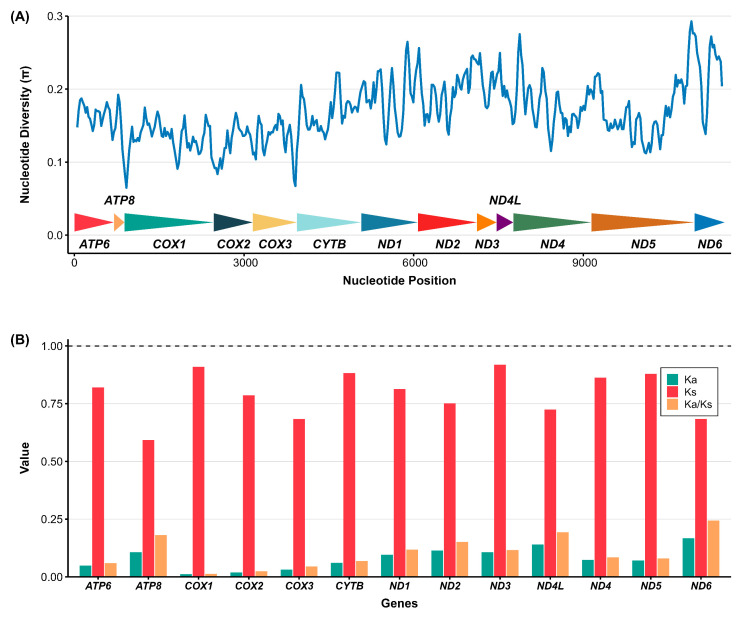
Sequence variability and selection patterns of 13 mitochondrial PCGs among Sisoridae. (**A**) Nucleotide diversity (π) calculated from the concatenated alignment of 13 PCGs from 48 Sisoridae sequences; (**B**) Ka, Ks, and Ka/Ks values calculated from separate orthologous PCG alignments for the same taxa.

PCG-level nucleotide diversity (π), calculated from the concatenated alignment of 13 mitochondrial PCGs from 48 Sisoridae sequences, is shown in Figure 4A. All 48 sequences were retained, and detailed alignment matrix statistics are provided in Table S2. The π values differed among mitochondrial PCGs, indicating gene-specific differences in PCG-level nucleotide diversity. Relatively high nucleotide diversity was observed in regions corresponding to *ND1*, *ND2*, *ND3*, *ND5*, and *ND6*, whereas lower diversity was mainly detected in parts of *ATP8*, *COX1*, and *COX3*. The highest peak occurred near the *ND5*-*ND6* region, where these genes contained relatively abundant variable sites.

The Ka, Ks, and Ka/Ks values of the 13 PCGs are shown in Figure 4B. All PCGs had Ka/Ks ratios below 1. Among the 13 PCGs, *ND6* showed the highest Ka/Ks ratio, followed by *ND4L* and *ATP8*, whereas *COX1* had the lowest Ka/Ks ratio.

### 3.5. Phylogenetic Analysis

**Figure 5:**
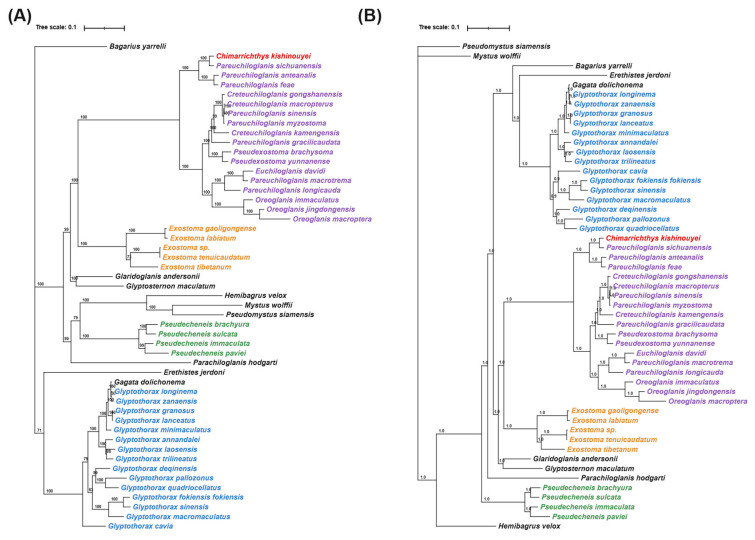
Phylogenetic placement of *C. kishinouyei* within Sisoridae inferred from 13 PCGs and two rRNA genes. (**A**) Maximum likelihood tree; (**B**) Bayesian inference tree. Tree scale is in substitutions per site. Major taxon groups are indicated by colored tip labels: *Pseudecheneis* (green), *Glyptothorax* (blue), *Exostoma* (orange), and the glyptosternoid group (purple); *C. kishinouyei* is shown in red.

Phylogenetic relationships within Sisoridae were inferred using the concatenated dataset of 13 PCGs and two rRNA genes under ML and BI methods. The ML and BI trees showed largely congruent topologies, with strong support for most major nodes (Figure 5). The analyzed Sisoridae species were clearly separated from the outgroup taxa, and several major lineages were recovered, including *Pseudecheneis*, *Glyptothorax*, *Exostoma*, and the glyptosternoid group containing *Pareuchiloglanis*, *Oreoglanis*, *Euchiloglanis*, *Pseudexostoma*, and *Creteuchiloglanis*.

In both phylogenetic trees, *C. kishinouyei* was nested within the glyptosternoid clade and formed a sister relationship with *Pareuchiloglanis sichuanensis*. This relationship received strong support in both analyses, with a bootstrap value of 100 in the ML tree and a posterior probability of 1.00 in the BI tree. The clade comprising *C. kishinouyei* and *P. sichuanensis* was further grouped with other *Pareuchiloglanis* species, including *P. feae* and *P. anteanalis*. *C. kishinouyei* did not cluster directly with *Euchiloglanis davidi* in either tree.

## 4. Discussion

The present study uses mitochondrial gene evidence from 13 PCGs and two rRNA genes to assess the phylogenetic placement of *C. kishinouyei* within Sisoridae. This focus is important because previous morphology- and marker-based studies have clarified major sisorid relationships, but several glyptosternoid affinities remain unresolved [8,9,10]. By adding *C. kishinouyei* to this mitochondrial gene dataset, the study provides a direct mitochondrial test of its affinity relative to *Pareuchiloglanis*, *Euchiloglanis*, and other glyptosternoid genera [1,2].

The conserved mitogenomic organization of *C. kishinouyei* supports this phylogenetic comparison. The genome contains the typical vertebrate mitochondrial gene set and lacks major rearrangements, indicating that its sequence data are directly comparable with other sisorid mitogenomes [1,3]. Similar conserved architectures have also been reported in recent comparative mitogenomic studies of freshwater fishes, where gene content, nucleotide composition, and mitochondrial phylogenies were used together to interpret lineage relationships [4,5,6].

The compositional and structural features of the mitogenome are consistent with broader mitochondrial patterns rather than species-specific abnormalities. The observed codon usage bias is likely associated with base composition, a common feature of animal mitochondrial genomes [19]. Likewise, the absence of a typical DHU arm in tRNA-Ser(GCU) is consistent with a widespread metazoan mitochondrial tRNA pattern and supports the functional completeness of the assembled genome [31].

The PCG-level nucleotide diversity and Ka/Ks analyses provide additional context for interpreting mitochondrial sequence evolution within Sisoridae. Ka/Ks ratios below 1 indicate that the 13 PCGs are mainly constrained by purifying selection, consistent with the essential role of mitochondrial PCGs in oxidative phosphorylation [32]. Differences among genes should therefore be interpreted as variation in functional constraint and phylogenetic informativeness, rather than as simple differences in overall evolutionary rate.

The phylogenetic analyses provide the central evidence of this study. Both ML and BI analyses placed *C. kishinouyei* within the glyptosternoid lineage and recovered a strongly supported sister relationship with *P. sichuanensis*. This result is noteworthy because *C. kishinouyei* was historically treated as *E. kishinouyei* and is now recognized within *Chimarrichthys* [11,12]. The absence of direct clustering with *E. davidi* suggests that relationships among *Chimarrichthys*, *Pareuchiloglanis*, and *Euchiloglanis* are more complex than implied by historical taxonomic treatments.

Nevertheless, the present result should be interpreted as mitochondrial phylogenetic evidence, not as a basis for formal taxonomic revision. Mitochondrial genomes represent a single maternally inherited marker system and may differ from species trees when incomplete lineage sorting, introgression, or rapid diversification has occurred [33]. The recent classification framework for Sisoridae and broader syntheses of siluriform taxonomy provide an important context for this interpretation [14,34,35]. Future studies combining mitochondrial genomes, nuclear genomic data, morphology, ecology, and broader taxon sampling will be needed to clarify generic boundaries and evolutionary relationships among these glyptosternoid catfishes [13,36].

## 5. Conclusions

This study provides a phylogenetic assessment of *C. kishinouyei* within Sisoridae based on 13 mitochondrial PCGs and two rRNA genes. The conserved mitogenome structure supports comparison with other sisorid taxa, and these phylogenetic analyses placed *C. kishinouyei* within the glyptosternoid lineage, with a closer mitochondrial affinity to *P. sichuanensis* than to *E. davidi*. These findings clarify the mitochondrial placement of *C. kishinouyei* and provide a concise framework for future integrative studies of glyptosternoid catfish relationships.

## Figures and Tables

**Table 1:** Nucleotide composition and strand skew of major mitogenomic regions in *Chimarrichthys kishinouyei*.

	A%	T%	G%	C%	A + T%	AT-Skew	GC-Skew
Whole mitogenome	31.79	25.48	15.26	27.47	57.27	0.11	−0.29
PCGs	30.23	27.24	14.77	27.76	57.47	0.05	−0.31
rRNAs	34.54	21.28	19.21	24.97	55.82	0.24	−0.13
tRNAs	29.02	27.29	22.42	21.27	56.31	0.03	0.03
D-loop	32.14	30.12	15.68	22.06	62.26	0.03	−0.17

## Data Availability

The genome sequence data supporting this study are openly available in GenBank of NCBI at https://www.ncbi.nlm.nih.gov (accessed on 26 June 2026) under the accession number PX134977. The associated BioProject, SRA, and Biosample numbers are PRJNA1300963, SRR34848407, and SAMN50431664, respectively.

## Supplementary Materials

The following supporting information can be downloaded at https://www.mdpi.com/article/10.3390/genes17070749/s1, Table S1: Information of species used in the phylogenetic analysis; Table S2: Alignment matrix statistics for PCG-level nucleotide diversity and Ka/Ks analyses; Table S3: Best-fit partition schemes and substitution models selected for phylogenetic analyses; Table S4: Mitochondrial genome annotation of *Chimarrichthys kishinouyei*; Figure S1: Predicted secondary structures of the 22 mitochondrial tRNAs in *Chimarrichthys kishinouyei*.
