# Supplementary material for: Mitogenomic Evidence for the Phylogenetic Placement of Chimarrichthys kishinouyei Within Sisoridae
Source: Genes (Basel). 2026 Jun 29;17(7):749. doi: 10.3390/genes17070749 (PMC13409716; doi:10.3390/genes17070749)
Supplement: Supplementary file 1 [file genes-17-00749-s001.zip › Table S1.pdf]

Table S1. Information of species used in the phylogenetic analysis.

Accession Number	Species	Family	Length	AT%
PP922174.1	*Hemibagrus velox*	Bagridae	16,511	58.1
PV815766.1	*Mystus wolffii*	Bagridae	16,573	58.9
PV946889.1	*Pseudomystus siamensis*	Bagridae	16,528	56.9
AP012012.1	*Erethistes jerdoni*	Sisoridae	16,632	56.5
KP872696.1	*Pseudexostoma brachysoma*	Sisoridae	16,583	54.6
MK617319.1	*Pareuchiloglanis myzostoma*	Sisoridae	16,584	54.9
MN082046.1	*Glyptothorax deqinensis*	Sisoridae	16,542	57.4
MN082047.1	*Pseudecheneis immaculata*	Sisoridae	16,432	58.5
MW715684.1	*Parachiloglanis hodgarti*	Sisoridae	16,511	58.6
NC_018769.1	*Glyptothorax fokiensis fokiensis*	Sisoridae	16,530	58.4
NC_021596.1	*Gagata dolichonema*	Sisoridae	16,538	57.1
NC_021597.1	*Glyptosternon maculatum*	Sisoridae	16,539	58.2
NC_021600.1	*Glaridoglanis andersonii*	Sisoridae	16,532	55.9
NC_021601.1	*Exostoma labiatum*	Sisoridae	16,542	56.8
NC_021603.1	*Pareuchiloglanis gracilicaudata*	Sisoridae	16,588	54.9
NC_021604.1	*Pseudexostoma yunnanense*	Sisoridae	16,598	54.6
NC_021605.1	*Pseudecheneis sulcata*	Sisoridae	16,474	58.6
NC_021606.1	*Bagarius yarrelli*	Sisoridae	16,503	56.8
NC_021607.1	*Oreoglanis macroptera*	Sisoridae	16,568	55.3
NC_021608.1	*Glyptothorax trilineatus*	Sisoridae	16,539	57.1
NC_024434.1	*Pareuchiloglanis sinensis*	Sisoridae	16,593	54.8
NC_024672.1	*Glyptothorax sinensis*	Sisoridae	16,531	58.3
NC_028509.1	*Creteuchiloglanis macropterus*	Sisoridae	16,592	54.9
NC_028511.1	*Oreoglanis immaculatus*	Sisoridae	16,576	55.7
NC_028512.1	*Oreoglanis jingdongensis*	Sisoridae	16,569	55.1
NC_028513.1	*Pareuchiloglanis anteanalis*	Sisoridae	16,554	56.6
NC_028514.1	*Pareuchiloglanis longicauda*	Sisoridae	16,535	55.7
NC_028515.1	*Pareuchiloglanis macrotrema*	Sisoridae	16,570	56.2
NC_028516.1	*Creteuchiloglanis gongshanensis*	Sisoridae	16,586	54.8
NC_029709.1	*Glyptothorax zanaensis*	Sisoridae	16,537	57.2
NC_034921.1	*Glyptothorax cavia*	Sisoridae	16,529	57.1
NC_039561.1	*Glyptothorax macromaculatus*	Sisoridae	16,535	57.5
NC_039702.1	*Glyptothorax laosensis*	Sisoridae	16,539	57.2
NC_039894.1	*Glyptothorax granosus*	Sisoridae	16,540	57
NC_039895.1	*Glyptothorax lanceatus*	Sisoridae	16,541	57.1
NC_039896.1	*Glyptothorax longinema*	Sisoridae	16,537	57
NC_042210.1	*Euchiloglanis davidi*	Sisoridae	16,569	56.4
NC_045213.1	*Creteuchiloglanis kamengensis*	Sisoridae	16,589	55.5
NC_045214.1	*Glyptothorax annandalei*	Sisoridae	16,541	56.9
NC_056351.1	*Exostoma gaoligongense*	Sisoridae	16,529	56.7
NC_059805.1	*Pareuchiloglanis sichuanensis*	Sisoridae	16,774	57.2
NC_061363.1	*Pareuchiloglanis feae*	Sisoridae	16,863	56.3
NC_065342.1	*Exostoma tibetanum*	Sisoridae	16,528	54.8
NC_065343.1	*Exostoma tenuicaudatum*	Sisoridae	16,533	55.2
NC_072245.1	*Glyptothorax pallozonus*	Sisoridae	16,542	59.2
NC_086847.1	*Pseudecheneis paviei*	Sisoridae	16,529	59.8
NC_088503.1	*Glyptothorax quadriocellatus*	Sisoridae	16,544	58.2
OK326860.1	*Exostoma* sp.	Sisoridae	16,529	55.2
OK329966.1	*Glyptothorax minimaculatus*	Sisoridae	16,536	57.1
PX134977.1	*Chimarrichthys kishinouyei*	Sisoridae	16,718	57.3
PZ055035.1	*Pseudecheneis brachyura*	Sisoridae	16,456	58.7
