# Supplementary material for: Mitogenomic Evidence for the Phylogenetic Placement of Chimarrichthys kishinouyei Within Sisoridae
Source: Genes (Basel). 2026 Jun 29;17(7):749. doi: 10.3390/genes17070749 (PMC13409716; doi:10.3390/genes17070749)
Supplement: Supplementary file 1 [file genes-17-00749-s001.zip › Table S4.pdf]

Table S4. Mitochondrial genome annotation of *Chimarrichthys kishinouyei*.

Genes	Position		Length	Codon		Strand
	From	To		Start	Stop	
$tRNA^{Phe}$	1	71	71			H
12S rRNA	72	1,023	952			H
$tRNA^{Val}$	1,024	1,095	72			H
16S rRNA	1,096	2,798	1,703			H
$tRNA^{Leu}$	2,799	2,873	75			H
ND1	2,876	3,844	969	ATG	TAG	H
$tRNA^{Ile}$	3,862	3,933	72			H
$tRNA^{Gln}$	3,933	4,003	71			L
$tRNA^{Met}$	4,004	4,072	69			H
ND2	4,074	5,118	1,045	ATG	T--	H
$tRNA^{Trp}$	5,119	5,188	70			H
$tRNA^{Ala}$	5,191	5,259	69			L
$tRNA^{Asn}$	5,261	5,333	73			L
$tRNA^{Cys}$	5,365	5,431	67			L
$tRNA^{Tyr}$	5,432	5,502	71			L
COX1	5,504	7,084	1,581	GTG	TAG	H
$tRNA^{Ser}$	7,051	7,121	71			L
$tRNA^{Asp}$	7,126	7,195	70			H
COX2	7,210	7,897	688	ATG	T--	H
$tRNA^{Lys}$	7,898	7,969	72			H
ATP8	7,971	8,159	189	ATG	TAA	H
ATP6	8,129	8,812	684	ATG	TAA	H
COX3	8,812	9,595	784	ATG	T--	H
$tRNA^{Gly}$	9,596	9,665	70			H
ND3	9,666	10,014	349	GTG	T--	H
$tRNA^{Arg}$	10,015	10,086	72			H
ND4L	10,090	10,383	294	ATG	TAA	H
ND4	10,377	11,760	1,384	ATG	T--	H
$tRNA^{His}$	11,761	11,830	70			H
$tRNA^{Ser}$	11,831	11,898	68			H
$tRNA^{Leu}$	11,903	11,975	73			H
ND5	11,976	13,802	1,827	ATG	TAA	H
ND6	13,799	14,314	516	ATG	TAG	L
$tRNA^{Glu}$	14,316	14,384	69			L
CYTB	14,388	15,525	1,138	ATG	T--	H
$tRNA^{Thr}$	15,526	15,602	77			H
$tRNA^{Pro}$	15,757	15,825	69			L
D-loop	15,826	16,718	893			
