# Supplementary material for: Mitogenomic Evidence for the Phylogenetic Placement of Chimarrichthys kishinouyei Within Sisoridae
Source: Genes (Basel). 2026 Jun 29;17(7):749. doi: 10.3390/genes17070749 (PMC13409716; doi:10.3390/genes17070749)
Supplement: Supplementary file 1 [file genes-17-00749-s001.zip › Table S3.pdf]

Table S3. Best-fit partition schemes and substitution models selected for phylogenetic analyses.

Partition	Subset partitions	ML model	BI model
P1	(atp6_mafft_NT_removed_chars_fas_gb_codon1)	GTR+F+I+G4	GTR+F+I+G4
P2	(atp6_mafft_NT_removed_chars_fas_gb_codon2_cytb_mafft_NT_removed_chars_fas_gb_codon2_nad1_mafft_NT_removed_chars_fas_gb_codon2)	K3Pu+F+R3	GTR+F+I+G4
P3	(atp6_mafft_NT_removed_chars_fas_gb_codon3_nad1_mafft_NT_removed_chars_fas_gb_codon3_nad3_mafft_NT_removed_chars_fas_gb_codon3_nad4_mafft_NT_removed_chars_fas_gb_codon3)	TIM3+F+I+R4	GTR+F+I+G4
P4	(atp8_mafft_NT_removed_chars_fas_gb_codon1_cytb_mafft_NT_removed_chars_fas_gb_codon1)	TVM+G4	GTR+G4
P5	(atp8_mafft_NT_removed_chars_fas_gb_codon2_cox3_mafft_NT_removed_chars_fas_gb_codon1)	TIM2+I+G4	SYM+I+G4
P6	(atp8_mafft_NT_removed_chars_fas_gb_codon3)	HKY+F+G4	HKY+F+G4
P7	(cox1_mafft_NT_removed_chars_fas_gb_codon1_cox2_mafft_NT_removed_chars_fas_gb_codon1)	GTR+F+I+G4	GTR+F+I+G4
P8	(cox1_mafft_NT_removed_chars_fas_gb_codon2_cox2_mafft_NT_removed_chars_fas_gb_codon2_cox3_mafft_NT_removed_chars_fas_gb_codon2)	TPM3u+F+I+R3	GTR+F+I+G4
P9	(cox1_mafft_NT_removed_chars_fas_gb_codon3)	GTR+F+I+R3	GTR+F+I+G4
P10	(cox2_mafft_NT_removed_chars_fas_gb_codon3_cox3_mafft_NT_removed_chars_fas_gb_codon3_nad4L_mafft_NT_removed_chars_fas_gb_codon3)	TN+F+I+R3	GTR+F+I+G4
P11	(cytb_mafft_NT_removed_chars_fas_gb_codon3)	GTR+F+I+R3	GTR+F+I+G4
P12	(nad1_mafft_NT_removed_chars_fas_gb_codon1)	GTR+F+I+G4	GTR+F+I+G4
P13	(nad2_mafft_NT_removed_chars_fas_gb_codon1_nad5_mafft_NT_removed_chars_fas_gb_codon1)	GTR+F+I+R4	GTR+F+I+G4
P14	(nad2_mafft_NT_removed_chars_fas_gb_codon2)	TPM3u+F+G4	GTR+F+G4
P15	(nad2_mafft_NT_removed_chars_fas_gb_codon3)	TN+F+I+R3	GTR+F+I+G4
P16	(nad3_mafft_NT_removed_chars_fas_gb_codon1)	TVM+F+I+G4	GTR+F+I+G4
P17	(nad3_mafft_NT_removed_chars_fas_gb_codon2_nad4_mafft_NT_removed_chars_fas_gb_codon2_nad5_mafft_NT_removed_chars_fas_gb_codon2)	TIM3+F+I+G4	GTR+F+I+G4
P18	(nad4L_mafft_NT_removed_chars_fas_gb_codon1)	GTR+I	GTR+I
P19	(nad4L_mafft_NT_removed_chars_fas_gb_codon2)	TPM3u+F+I	F81+F+I
P20	(nad4_mafft_NT_removed_chars_fas_gb_codon1)	GTR+F+I+G4	GTR+F+I+G4
P21	(nad5_mafft_NT_removed_chars_fas_gb_codon3)	TIM2+F+I+R3	GTR+F+I+G4
P22	(nad6_mafft_NT_removed_chars_fas_gb_codon1)	TIM2+F+I+R2	GTR+F+I+G4
P23	(nad6_mafft_NT_removed_chars_fas_gb_codon2)	TIM+F+R3	GTR+F+I+G4

Partition	Subset partitions	ML model	BI model
P24	(nad6_mafft_NT_removed_chars_fas_gb_codon3)	GTR+F+R3	GTR+F+I+G4
P25	(rrnL_muscle_trimAl)	GTR+F+R3	GTR+F+I+G4
P26	(rrnS_muscle_trimAl)	GTR+F+R4	GTR+F+I+G4
