# Supplementary material for: Mitogenomic Evidence for the Phylogenetic Placement of Chimarrichthys kishinouyei Within Sisoridae
Source: Genes (Basel). 2026 Jun 29;17(7):749. doi: 10.3390/genes17070749 (PMC13409716; doi:10.3390/genes17070749)
Supplement: Supplementary file 1 [file genes-17-00749-s001.zip › Table S2.pdf]

Table S2. Alignment matrix statistics for PCG-level nucleotide diversity and Ka/Ks analyses.

**Dataset**	**Aligned sequences**	**Alignment length (bp)**	Gap/missing cells across alignment matrix	Gap/missing cells (% of alignment matrix)	**Ambiguous N**
concatenated_13PCGs_for_pi	48	11499	5190	0.94	0
atp6	48	699	852	2.54	0
atp8	48	186	729	8.17	0
cox1	48	1578	1062	1.40	0
cox2	48	690	51	0.15	0
cox3	48	783	0	0.00	0
cytb	48	1137	0	0.00	0
nad1	48	1002	1587	3.30	0
nad2	48	1044	0	0.00	0
nad3	48	348	3	0.02	0
nad4	48	1383	99	0.15	0
nad4L	48	294	42	0.30	0
nad5	48	1824	6	0.01	0
nad6	48	531	759	2.98	0
