# Supplementary material for: Mitogenomic Evidence for the Phylogenetic Placement of Chimarrichthys kishinouyei Within Sisoridae
Source: Genes (Basel). 2026 Jun 29;17(7):749. doi: 10.3390/genes17070749 (PMC13409716; doi:10.3390/genes17070749)
Supplement: Supplementary file 1 [file genes-17-00749-s001.zip › Figure S1.pdf]

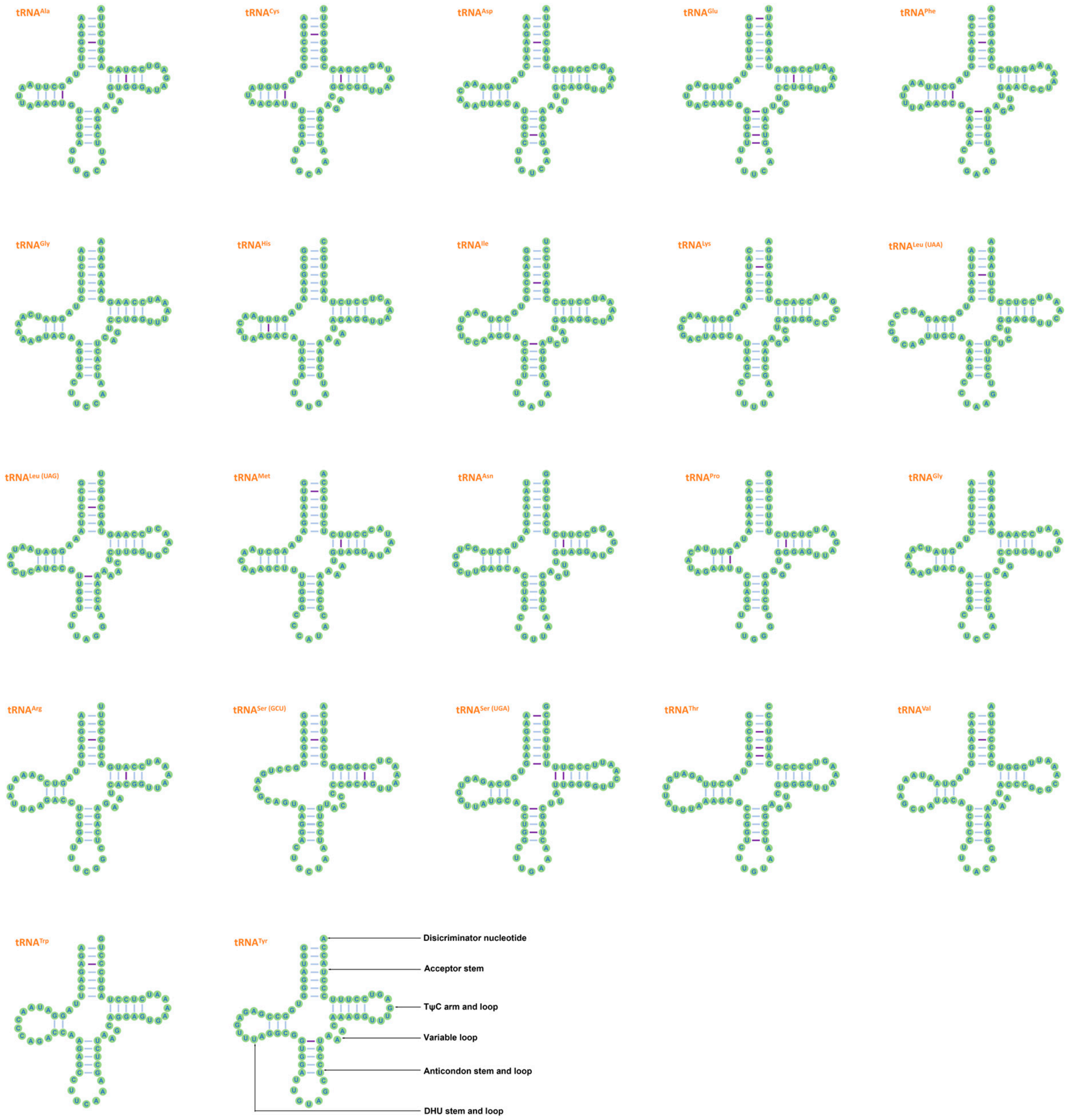

Figure S1. Predicted secondary structures of the 22 mitochondrial tRNAs in *Chimarrichthys kishinouyei*.
